# A Novel Approach to Femoral Cartilage Repair: Episealer Twin Implantation Case Report

**DOI:** 10.1155/cro/5554169

**Published:** 2025-12-18

**Authors:** Marwan Ibrahim, Ivan Wong

**Affiliations:** ^1^ Department of Surgery, Milton Keynes University Hospital NHS Foundation Trust, Milton Keynes, UK; ^2^ Oxford Foundation School, Oxford, UK; ^3^ Faculty of Medicine, Dalhousie University, Halifax, Nova Scotia, Canada, dal.ca; ^4^ Division of Orthopaedic Surgery, Dalhousie University, Halifax, Nova Scotia, Canada, dal.ca; ^5^ Division of Orthopaedic Surgery, Nova Scotia Health, Halifax, Nova Scotia, Canada, nshealth.ca

**Keywords:** case report, chondral, Episealer, femoral condyle, knee, osteochondral

## Abstract

Episealer metal implants have recently been gaining attention, offering treatment for focal chondral and osteochondral lesions, particularly in the knee joint. These patient‐specific implants are precisely made using a detailed MRI analysis of the lesions, bridging a critical gap in the treatment of younger patients with challenging degenerative lesions. This innovative approach provides safe, predictable, and effective measures to preserve the function of the knee joint and maintain its native structure. In this case report, we describe the surgical outcomes of an Episealer femoral twin implantation, focusing on treating a lesion spanning the lateral condyle and trochlear region of the femur. This was performed on a 44‐year‐old patient complaining of an 8‐month history of knee pain after a nontraumatic injury. The patient was found to have a Grade 4 osteochondral lesion on the lateral femoral condyle and elected to receive the Episealer twin metal implant. Postoperative measurements showed an improvement in the range of movement and strength. The patient also reported improvement in pain, knee functionality, and overall quality of life. In conclusion, detailed MRI analysis made it possible to design patient‐specific implants, effectively addressing the gap in the treatment of younger patients with focal degenerative lesions.

## 1. Introduction

Focal osteochondral and chondral lesions of the knee joint are shown to be challenging and debilitating for patients below the age of 50 [1]. While the treatment of focal osteochondral and chondral lesions is readily conducted in older adults through the use of biological treatments, such as autologous chondrocyte transplantation, or either total or unicompartmental knee arthroplasty, a gap is seen in the treatment of middle‐aged patients who do not meet the inclusion criteria of these treatment options [2–4]. Such lesions present a challenge in younger patients as they may lead to osteoarthritis and consequently reduce their quality of life with age. Several procedures have been developed in an attempt to better treat middle‐aged patients diagnosed with focal lesions in the knee joint, such as osteochondral allograft (OCA)/autograft transfer and microfracturing [5–7]. These procedures consider the patient′s age to determine the most suitable approach, the extent of damage to the articulating cartilage, and the severity of symptoms [6]. While these treatments provide additional surgical options for the middle‐aged population, microfracture has shown variable and often declining long‐term success rates [7]. In contrast, procedures such as osteochondral autograft transfer system (OATS) and OCA have demonstrated good outcomes in appropriately selected patients [5], although none of these techniques have achieved universal acceptance as the definitive standard of care [6].

In this case report, we present the use of a newly emerging intervention, metal resurfacing implants, to address the gap seen in the treatment of middle‐aged patients with focal osteochondral and chondral lesions. Metal resurfacing implants are a newly emerging approach that addresses articulating cartilage pathologies, developed explicitly for treating focal lesions within the medial femoral condyle, lateral femoral condyle, or the femoral trochlea area [8, 9]. Episealer are patient‐specific resurfacing implants designed to treat these types of lesions. The Episealer implants are uniquely created according to the lesion size and anatomical location [10, 11]. Using magnetic resonance imaging (MRI), knees are assessed for all aspects of chondral and osteochondral defects, and precise measurements are taken specific to the patient′s lesion. These measurements are used to engineer patient‐specific surgical instruments and implants [10, 11]. This case report illustrates the postsurgical outcomes of the Episealer lateral femoral twin implantation on a patient with a Grade 4 cartilage lesion. The Episealer twin implantation is suitable for treating cartilage lesions affecting condylar and trochlear areas.

## 2. Case Presentation

In this case report, we present a 44‐year‐old female patient (height, 5 ft 9 in. [180 cm]; body mass index, 32.5 kg/m^2^) who had a nontraumatic injury to the right knee. The injury occurred due to a sudden twisting of the knee during a social event. The patient reported hearing a popping sound and an immediate inability to weight bear with extreme pain and swelling. Eight months after the incident, the patient was still experiencing constant soreness, which worsened throughout the day. The pain increased significantly with any unpredicted movement or touch of the right knee. The patient also reported sudden sharp pain coming from inside the knee joint. This pain occurred 1–2 times a day.

The patient initially underwent a comprehensive course of nonoperative management, including regular paracetamol and supervised physiotherapy sessions focused on quadriceps strengthening, range‐of‐motion, and functional rehabilitation exercises. Despite completing 6 months of structured physiotherapy, there was no significant improvement in pain levels or functional capacity.

Past surgical history includes previous anterior cruciate ligament (ACL) reconstruction and meniscal repair on the contralateral knee. The patient′s past medical history is noncontributory, except for osteoarthritis in the contralateral knee. No significant family history was reported. The patient enjoys walking and recreational swimming. Engagement levels in those activities decreased since the incident due to pain in the affected knee. The patient does not smoke or drink alcohol.

### 2.1. Preoperative Clinical Findings

On inspection, the patient did not have any gait abnormalities, valgus, or varus deformities. Mild to moderate pain was reported in the right knee when walking and relieved with rest. The right knee had obvious signs of joint swelling compared to the left knee. No tenderness or bony abnormalities were reported on palpation of the right knee. However, pain was elicited on palpating the medial collateral ligament (MCL) along with valgus stress, and slight discomfort was observed on passive movement of the affected knee. The patient was able to flex the right knee up to 115°, but extension was limited to 5°. Examination of Lachman′s and anterior drawer tests were limited due to guarding.

**Figure 1:**
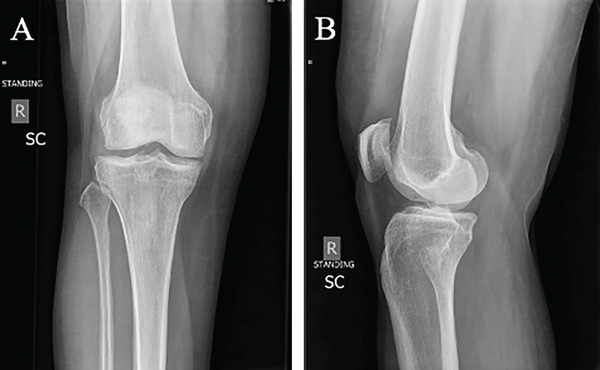
Preoperative radiograph of the right knee showing the osteochondral defect at the lateral femoral condyle from the frontal view (A) and more predominantly from the lateral view (B).

**Figure 2:**
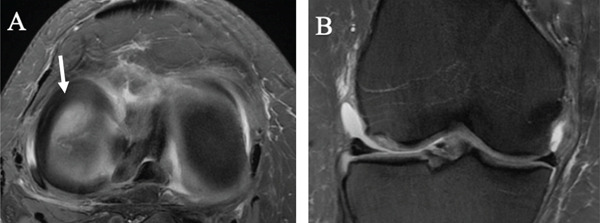
Preoperative magnetic resonance imaging of (A) axial and (B) coronal views of the right knee showing osteochondral impaction at the lateral femoral condyle (white arrow), with lateral meniscal tear at the posterior horn attachment and underlying bone marrow edema.

**Figure 3:**
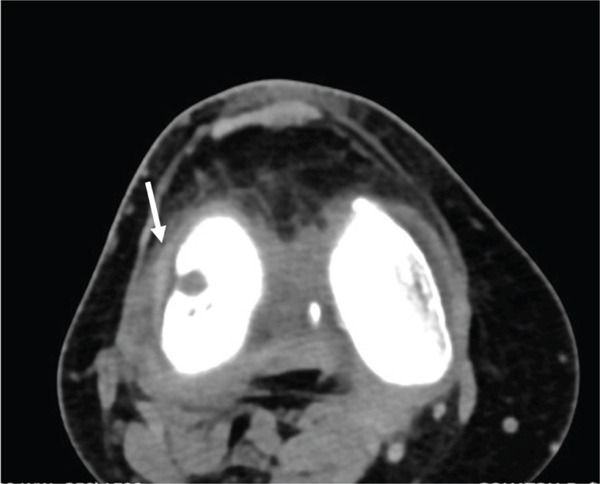
Preoperative computed tomography views of the right knee further confirm the osteochondral defect at the lateral aspect of the lateral femoral condyle (white arrow), measuring approximately 1.4 × 1.2 × 0.5 cm.

Diagnostic imaging included radiographs, MRI, and computed tomography (CT) with three‐dimensional (3D) reconstruction of the right knee. Following radiological evaluation, the patient was found to have a Grade 4 osteochondral lesion of the lateral femoral condyle with subchondral bone involvement, along with an ACL tear of the right knee (Figure 1). The MRI confirmed this defect and demonstrated a complex tear of the lateral meniscus predominantly at the posterior horn, moderate to large joint effusion, and evidence of an ACL tear (Figure 2). While MRI was the primary imaging modality for diagnosis and assessment of the cartilage and soft tissue structures, CT with 3D reconstruction was additionally performed to provide detailed evaluation of the bony architecture, accurately measure the defect, and aid in preoperative templating for the Episealer implant. The CT showed that the joint space and alignment were well maintained, with some tricompartmental osteophytes, and measured the osteochondral defect to be 1.4 cm (AP) × 1.2 cm (TR) × 0.5 cm (depth) (Figure 3).

### 2.2. Therapeutic Intervention

The patient elected to undergo surgery including right knee arthroscopy, ACL reconstruction with allograft, meniscectomy, and Episealer lateral femoral twin implantation to address the osteochondral defect of the lateral femoral condyle. The patient was thoroughly assessed to determine eligibility for the Episealer implant. Evaluating the patient′s eligibility is vital, as having an isolated focal lesion on femoral condyles and good overall health are crucial for this procedure. Eligibility criteria for this procedure include an isolated, well‐defined focal osteochondral lesion on a weight‐bearing area of the femoral condyle, lesion size between 1 and 4 cm^2^, intact or reconstructable surrounding structures, absence of generalized osteoarthritis, and failure of at least 6 months of nonoperative management [10]. The patient met all these criteria, with a 1.4 × 1.2 × 0.5 cm lesion, preserved joint alignment, and no diffuse degenerative changes. Alternative options such as microfracture, OATS/OCA, or autologous chondrocyte implantation (ACI) were considered; however, microfracture has limited long‐term efficacy in middle‐aged patients, OATS and OCA are more invasive with donor site or graft‐related challenges, and the patient was not a candidate for arthroplasty due to her age and activity level. Given these factors, the Episealer implant was selected as the most appropriate option to restore the focal defect and preserve native joint structures.

Using data obtained from MRI, a final report was created with precise lesion size and anatomical location measurements. Once the measurements are checked and approved by the operating surgeon, patient‐specific implant and corresponding instruments are manufactured. A printed 3D model of the condylar defect was created for preoperative planning and intraoperative assessment.

A diagnostic arthroscopy of the right knee was performed to evaluate intra‐articular structures, remove loose bodies, and assess the meniscal and chondral injuries. A partial lateral meniscectomy addressed the posterior horn tear, and ACL reconstruction using an allograft was carried out arthroscopically with standard tunnel preparation and graft fixation to restore stability.

**Figure 4:**
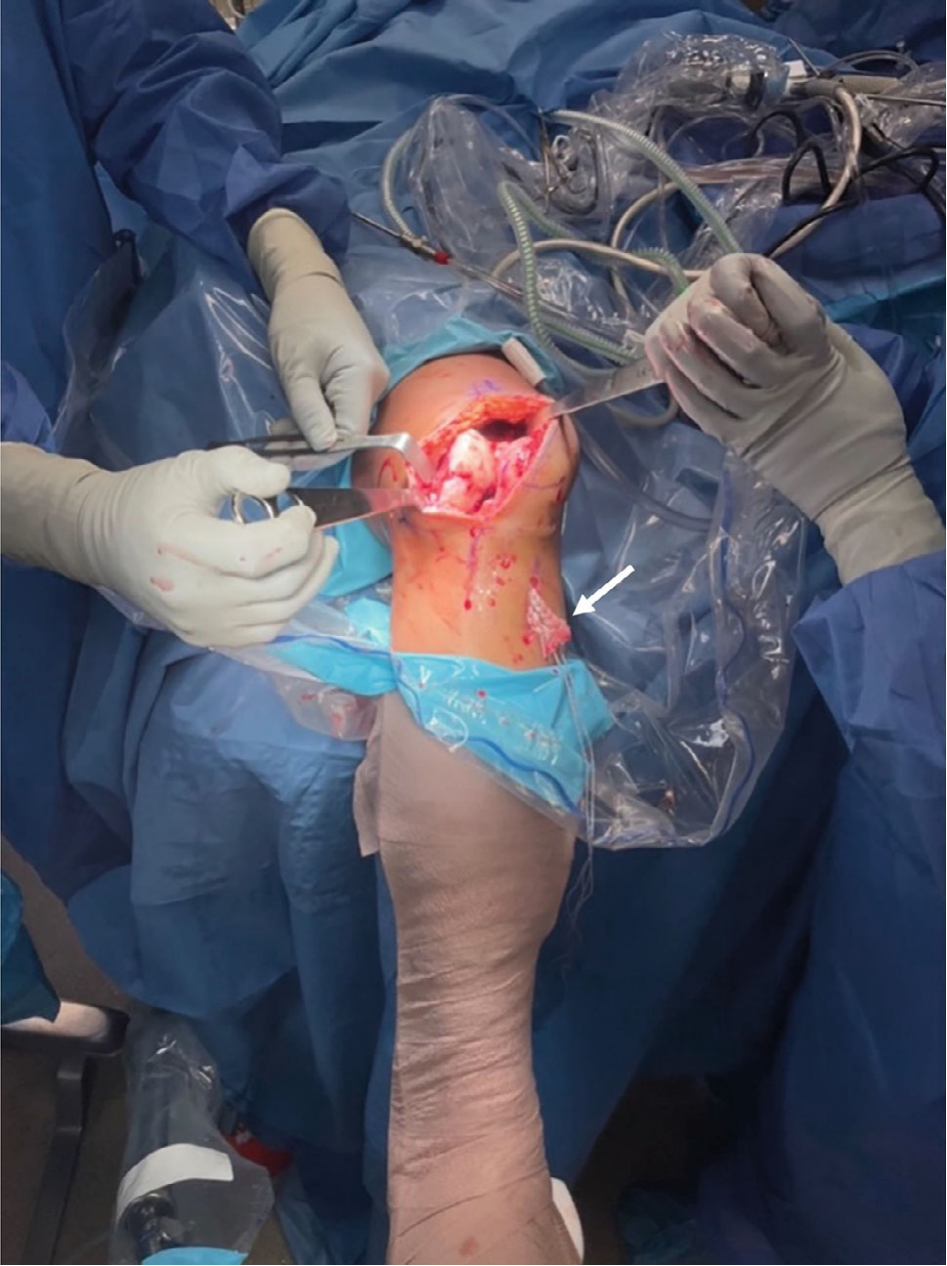
The patient is placed supine, and the surgical field is prepared. The patient′s leg is secured to the pneumatic positioner. The knee is flexed 90°, and a lateral parapatellar incision is made to gain clear access to degenerative lesions on the lateral femoral condyle. The ACL (white arrow) is passed through the tunnel and tensioned for insertion at the end of the procedure.

Subsequently, the lateral femoral condyle was exposed. The lesion was debrided, and the surrounding cartilage rim was prepared to create stable borders. A 4‐cm midline incision allowed placement of the Epiguide for accurate positioning, followed by stepwise drilling and milling under direct visualization to prepare the recipient site for the Episealer twin implant (Figure 4).

**Figure 5:**
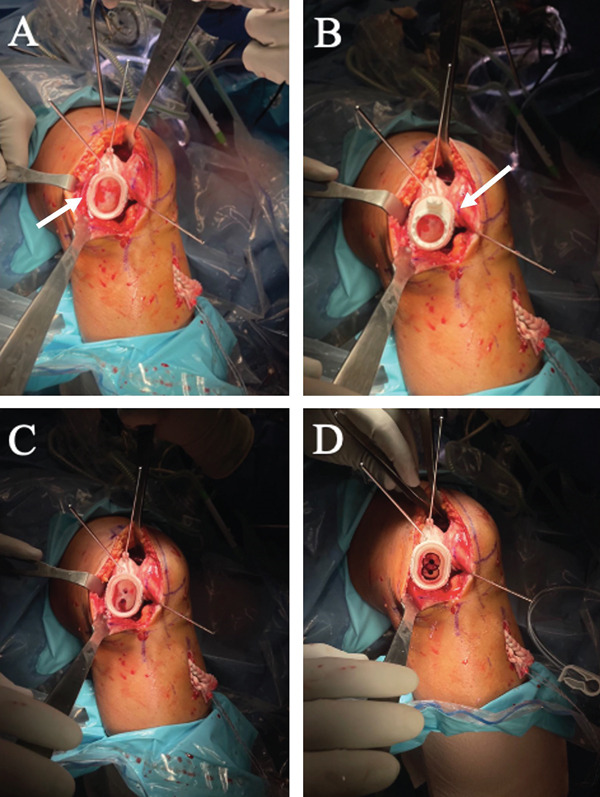
Episealer twin femoral implant procedure. (A) Epiguide (white arrow) is placed with its opening covering the lesions on the lateral condyle and trochlea. Three pins securely attach the Epiguide to the bone to avoid any movement while drilling. (B) An insert (white arrow) is mounted into the Epiguide. The insert comes in two orientations, with a 180° difference, to guide the drilling of the two holes. (C) The results of the first drilling procedure which creates two peg holes equal in depth and length. (D) The results of the final drilling steps which create two adjustment holes around the predrilled peg holes. Debris must be cleared from the peg holes to avoid loosening or migration of the Episealer metal implant. On completion, all tools around the holes will be removed for the final implantation of the Episealer.

**Figure 6:**
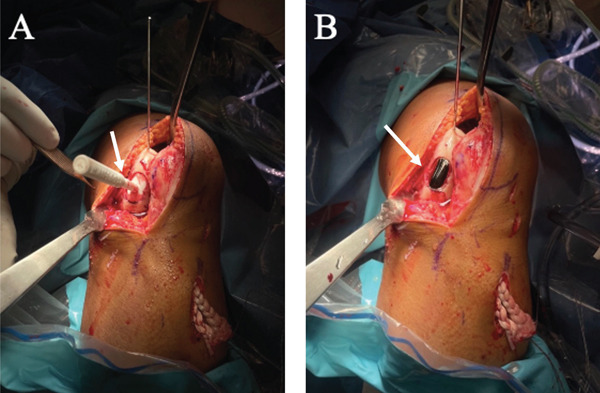
Final placement of the twin Episealer metal implant. (A) All tools and guides are removed around the hole. The depth and alignment are checked with the Epidummy (white arrow) before the final implantation. (B) The twin Episealer metal implant (white arrow) is in place with a 0.5–1 mm gap below the adjacent cartilage surfaces.

The Episealer twin implantation requires two holes along the femoral condyle. To achieve this, an Epiguide was used for the entire duration of the surgery to guide the drilling of both holes (Figure 5A). An insert is placed into the Epiguide (Figure 5B), which comes in two orientations, one for each hole (Figure 5C,D). The tool kit contains an Epidrill specifically designed for the Episealer twin implantation and an Epidummy, which is used to assess the distance between the two holes, their width, and depth. Upon completion of the drilling procedure, the Epiguide is removed, and the depth is checked for a final time using the Epidummy before the final implantation (Figure 6A). The Episealer is gently pressed down using finger pressure to fixate it in place. Once fixed, a hammer and an Epimandrel tap the Episealer into the femoral condyle. The position of the implant is evaluated and completed when there is a 0.5–1 mm gap between the Episealer and the adjacent cartilage surface (Figure 6B). Table 1 outlines the key steps of the Episealer metal implantation procedure.

**Table 1:** Surgical steps of the Episealer lateral femoral twin implantation.

1. The patient is placed supine under general anesthesia.
2. The affected knee is attached to a pneumatic holder and positioned at 90°.
3. A diagnostic scope is carried out for evaluation and debridement of the affected knee.
4. The Episealer twin implantation procedure starts with a 4‐cm midline incision to expose the lateral femoral condyle and prepare for the Epiguide placement.
5. The Epiguide is secured to the lateral femoral condyle using three surgical pins.
6. An insert is placed into the Epiguide.
7. The drilling socket is mounted onto the Epiguide.
8. The Epidrill is attached to an assigned surgical drill and adjusted for drilling in the clockwise direction.
9. The Epidrill is inserted into the drilling socket, and drilling starts to create two peg holes.
10. An Epidummy is used to assess the distance between the two peg holes and evaluate their width and depth.
11. The peg holes are used as a guide to scribe the chondral lesions around them and create two wider holes, known as the adjustment holes.
12. All tools are detached, and vigorous debridement is done to clear the area from any loose cartilage.
13. The Twin Episealer metal implant is placed with a 0.5–1 mm gap below the adjacent cartilage surfaces.

### 2.3. Follow‐Up and Outcomes

**Figure 7:**
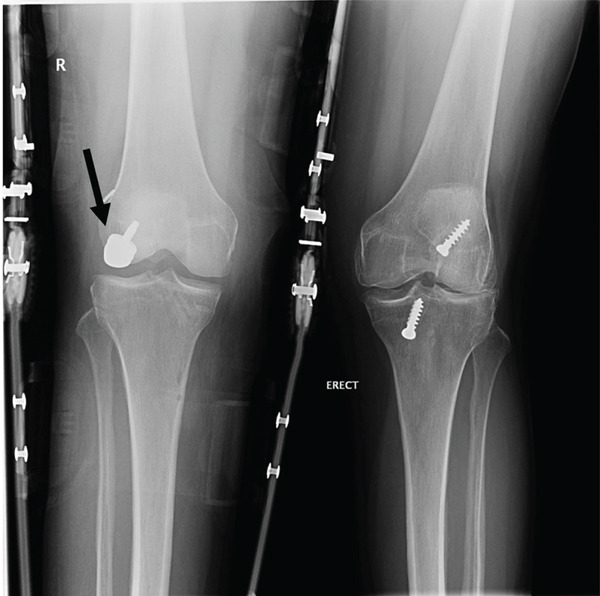
Postoperative anteroposterior view of the right and left knees. The right knee shows the Episealer twin metal implant well intact (black arrow), with good alignment, no joint effusion, and joint space preserved. The metallic components on both sides of the right knee are from the patient′s brace. The left knee shows the previously repaired left ACL.

The patient participated in a physiotherapy rehabilitation protocol specific to the Episealer implant. The protocol included partial weight bearing postoperative with full weight bearing achieved by 4–6 weeks unless otherwise restricted due to concurrent procedures. Following 6 weeks, they can begin low‐impact cardiovascular exercises, for example, swimming and biking, and gradually progress with strength improvements. This rehabilitation protocol was adhered to as reported by physiotherapy. The patient was seen clinically at 2‐week, 8‐week, and 6‐month postoperative time points. A radiograph was taken 2 and 8 weeks postoperatively, both of which showed satisfactory alignment, stable hardware, and no joint effusion (Figure 7). No further radiographs were obtained after 8 weeks; however, complete osseointegration of the implant is typically expected between 6 and 12 months postoperatively, as reported in the literature [10].

At 6 months, the patient demonstrated improved range of motion, with knee flexion increasing from 134° preoperatively to 146° and maintaining full extension. Strength also improved compared to preoperative measurements, with knee extension increasing from 74.2 to 85.1 lbs, gluteus medius from 64.9 to 73.5 lbs, and gluteus maximus from 59.2 to 66.4 lbs (Table 2).

**Table 2:** Comparison of right knee range of motion pre to postoperatively. Comparison of right knee strength pre to postoperatively. Gmed: gluteus medius; Gmax: gluteus maximus.

	**Preop**	**8-week postop**	**6-month postop**
Range of motion			
Flexion (degrees)	134	118	146
Extension (degrees)	0	0	0
Strength			
Flexion (lbs)	44.7	12	39.6
Extension (lbs)	74.2	23.5	85.1
Gmed (lbs)	64.9	60	73.5
Gmax (lbs)	—	59.2	66.4

Patient‐reported questionnaires showed improvements as well, with the IKDC increasing from 33.3 to 51.7, pain severity decreasing from 6/10 to 4/10, and function of the injured knee improving from 2/10 to 6/10. EQ‐5D‐5L domains improved across mobility, self‐care, and pain dimensions, while the EQ‐5D VAS decreased slightly from 72 to 62 postoperatively (Table 3).

**Table 3:** Patient‐reported outcome score measured preoperatively and 6 months postoperatively. IKDC, International Knee Documentation Committee Subjective Knee Evaluation Form.

	**Preoperatively**	**Postoperatively**
EQ‐5D‐5L		
• Mobility	3	2
• Self‐care	2	1
• Usual activities	4	3
• Pain/discomfort	4	3
• Anxiety/depression	1	1
EQ‐5D VAS	72	62
IKDC	33.3	51.7
Pain severity	6/10	4/10
Function of injured knee	2/10	6/10

Formal physiotherapy and outcome data were collected up to 6 months; however, the patient was followed clinically for 2 years, during which she consistently reported no pain, functional limitations, or other concerns. The 2‐year postoperative visit represented the final clinical follow‐up, while the 6‐month data were used for early postoperative assessment. No adverse events were reported.

## 3. Discussion

This case report demonstrates the surgical outcomes of an Episealer twin femoral implant used to treat a focal osteochondral lesion of the lateral femoral condyle. As described earlier in the manuscript, careful patient selection is essential for this joint‐preserving procedure. In this case, patient‐specific MRI‐based planning allowed precise implant design and placement to match the lesion′s size, depth, and location.

This case highlights the successful management of a focal Grade 4 osteochondral lesion using a twin Episealer femoral implant. In this case report, we present a 44‐year‐old female with an 8‐month history of right knee pain following a nontraumatic injury. Investigations revealed a Grade 4 osteochondral lesion on the lateral femoral condyle, a complex lateral meniscal tear, and an ACL tear. Analgesics and physiotherapy provided minimal relief. While arthroplasty was not appropriate given the patient′s age and activity level, other cartilage restoration options such as microfracture, OATS, and ACI were considered; however, based on the lesion size, location, and patient factors, the Episealer implant was deemed the most suitable option. The patient underwent Episealer twin femoral implantation with concurrent partial lateral meniscectomy and ACL reconstruction. Tables 2 and 3 show postoperative improvements in range of motion, strength, pain, and functional scores compared with preoperative measures.

Focal lesion resurfacing is gaining wider acceptance, with data showing clinical improvement and low failure rates [10]. Holz et al. reported significant improvements in KOOS and VAS scores at 1 and 2 years postoperatively in 80 patients treated with Episealer implants, with mean KOOS improving from 35 to 57 and 59 at 1 and 2 years, respectively [10]. Similar findings have been observed in smaller cohorts, with no evidence of disease progression or implant migration [12]. This case adds to the existing literature by describing the use of a twin Episealer implant to treat a lateral femoral condyle osteochondral lesion, a combination that is less frequently reported compared to the more common single medial condyle implants. It also includes 2‐year clinical follow‐up, with patient‐reported questionnaires up to 6 months postoperatively, with good functional outcomes and no complications.

The Episealer metal implants have been shown to exert chondroprotective effects on the surrounding articular cartilage [13]. No osteoarthritic changes have been observed postoperatively in appropriately selected patients [13]. The surrounding cartilage is supported by the accurately positioned implant, which helps reduce the progression of degenerative defects [14, 15]. These protective effects are largely attributed to the precise implantation technique and optimal implant depth of approximately 0.5 mm below the surrounding cartilage, which limits degenerative expansion [16, 17]. The undersurface and edges of the implant are coated with hydroxyapatite to promote osseointegration with the surrounding subchondral bone, thereby enhancing implant stability and indirectly supporting adjacent cartilage [13].

The safety and protective properties of the Episealer implant depend significantly on the strict inclusion criteria applied to patients [11]. This crucial process involves an extensive assessment of the patient′s medical history, previous injuries, and overall knee condition. In a study by Bollars et al., 27 patients were treated with a focal metal implant, nine of whom did not meet the inclusion criteria [17]. Cartilage degenerative changes were seen in seven of the nine patients, all of whom required total knee replacement surgery [17]. Another study by Holz et al. also used an inclusion criterion to select 80 patients for focal metal implant [10]. The rate of revision surgeries was reported to be 2.5% (2 out of 80 patients) [10]. This was due to the progression of osteoarthritis observed in one patient and the lack of pain improvement seen in the second patient postoperatively [10]. These results illustrate the importance of the appropriate selection of patients for an Episealer implant and how it significantly contributes to its protective efficacy and overall safety.

The use of the Episealer metal implant is rapidly advancing, with promising data showing its short‐ and medium‐term effectiveness. However, it is crucial to interpret this data cautiously due to the relatively short follow‐up periods reported in the current literature. Additional data need to be collected to gain a comprehensive understanding of the long‐term outcome of this approach. While generally reported as low, the failure and revision rates are inconsistent across different studies. This is primarily attributed to the reliance on a small sample size. Therefore, a larger patient cohort should be used to better understand resurfacing implants′ efficacy. This would help refine the selection criteria and ensure a high success rate.

Our patient reported positive feedback surrounding the outcome of her knee postoperatively. She offered, “Overall no regrets about the implant. My knee feels solid. I had some lingering spontaneous pain, but it did go away at the 7‐month mark. In the context of my age, stage of life and the complexity of my knee injury, I hope future surgeons take into account the whole patient when determining protocols and expectations for recovery and postoperative physio goals.” She reports continued improvements in her knee condition leading up to 1 year postoperatively.

Overall, the Episealer implant provides a promising solution for managing focal cartilage defects. Its advantages include a patient‐specific design that allows precise anatomical fit, preservation of native bone and joint structures, and avoidance of donor site morbidity or staged procedures associated with other techniques. Early postoperative weight bearing and favorable midterm outcomes with low failure rates have also been demonstrated [10]. Alternative surgical options include microfracture, which is cost‐effective but has limited long‐term durability [7]; ACI, which offers durable results but requires two surgeries and higher cost [18]; and the OATS, which provides immediate cartilage coverage but is restricted by lesion size and donor site availability [19]. OCA transplantation is another joint‐preserving alternative, offering mature hyaline cartilage restoration for larger lesions, but it is similarly expensive, limited by graft availability, and carries risks of immune response or incomplete graft integration [19].

A limitation of this case report is that the observed clinical improvements cannot be solely attributed to the Episealer implant, as concurrent ACL reconstruction and partial meniscectomy may have contributed to the overall outcome. Cost also remains a significant limitation, with implants typically exceeding $10,000, underscoring the need for careful patient selection and shared decision‐making [17].

In conclusion, Episealer implantation is a safe and effective joint‐preserving option for focal osteochondral lesions in appropriately selected patients. Detailed MRI analysis made it possible to design patient‐specific implants, effectively addressing the gap in the treatment of younger patients with focal degenerative lesions.

## Consent

The patient has given her informed consent for her clinical data to be used to publish this case report.

## Conflicts of Interest

Marwan Ibrahim—the author declares no known competing financial interests or personal relationships that could have appeared to influence the work reported in this paper. Ivan Wong—the author declares the following financial interests/personal relationships which may be considered as potential competing interests: Ivan Wong reports a relationship with DePuy Mitek Inc that includes consulting or advisory and speaking and lecture fees. Ivan Wong reports a relationship with Smith and Nephew Inc that includes consulting or advisory and speaking and lecture fees. Ivan Wong reports a relationship with CONMED Corp that includes consulting or advisory and speaking and lecture fees. Ivan Wong reports a relationship with Bioventus LLC that includes speaking and lecture fees. Ivan Wong is an editorial board member for the *American Journal of Sports Medicine* (*AJSM*) and *Arthroscopy: The Journal of Arthroscopic and Related Surgery* (*ARTH*) and *The HIVE Musculoskeletal Journal* and is a board or committee member for AANA, ISAKOS, and AAC. Ivan Wong is an editor for the *Orthopaedic Journal of Sports Medicine*.

## Funding

No funding was received for this manuscript.

## Data Availability

The data that support the findings of this study are available from the corresponding author upon reasonable request.
